# Development of an isotope dilution gas chromatography − mass spectrometry candidate reference measurement procedure for glucose in human serum

**DOI:** 10.1016/j.jmsacl.2025.04.005

**Published:** 2025-04-17

**Authors:** Komal Dahya, Heather C. Kuiper, Sarah W. Kingsley, Uliana Danilenko, Hubert W. Vesper

**Affiliations:** a Division of Laboratory Sciences, National Center for Environmental Health, Centers for Disease Control and Prevention, Atlanta, GA 30341, USA; b Battelle Memorial Institute, 2987 Clairmont Rd, Atlanta, GA 30329, USA

**Keywords:** Candidate Reference Measurement Procedure, Gas Chromatography, Mass Spectrometry, Glucose, Point of Care Testing

## Abstract

**Introduction:**

Diabetes is the seventh leading cause of death in the United States, impacting over 37 million people. Accurate glucose measurements are critical for effective diabetes management. A reliable candidate reference measurement procedure (cRMP) for assessing the analytical performance of glucose tests performed in patient care is essential for ensuring measurement accuracy.

**Methods:**

We have developed a gas chromatography-mass spectrometry (GC–MS)-based cRMP for glucose in human serum. In this procedure, glucose is measured as the aldononitrile acetate derivative and quantitated using a ^13^C_6_-glucose internal standard.

**Results:**

Analytical selectivity was achieved through chromatographic separation and monitoring the quantitation ion/confirmation ion ratios in samples. With bias ranging from −0.79 % to 0.67 % for eight levels of serum-based certified reference materials from the National Institute of Standards and Technology (NIST) and Laboratoire national de métrologie et d’essais (LNE) and total CVs of 1.11 %, 0.68 % and 0.74 % at the low, medium, and high glucose concentration levels, respectively, the cRMP provided excellent accuracy and precision. The calibration curve was linear throughout the 13.51–378.21 mg/dL [0.75–21 mmol/L] measurement range (R^2^ = 0.9999), with a mean slope of 270.73 (95 % CI, 270.19 to 271.27) and an intercept of 0.021 (95 % CI, −0.157 to 0.199). The limit of detection was 0.25 mg/dL (0.014 mmol/L) and the limit of quantitation was 0.83 mg/dL (0.046 mmol/L).

**Conclusion:**

The described GC–MS method, with metrological traceability to the International System of Units (SI), provides highly accurate and precise measurements of glucose in human serum.

## Highlights

• Highly accurate and precise reference measurement procedure for glucose, traceable to SI.
• Only continuous operating reference measurement procedure for glucose in the USA.
• Method can be used as a reference point to assess routine measurements and POCT devices.

## Introduction

Diabetes is a major public health concern [1]. Glucose, one of the main biomarkers for diabetes management, is measured in both healthcare and at-home settings, with the latter primarily using point-of-care testing (POCT) devices [2]. In both settings, accurate glucose measurements are critical for timely and appropriate glycemic control. Studies have reported inaccurate measurements using glucose monitoring devices typically used in healthcare settings [[3], [4], [5], [6], [7]]. One study indicated that the accuracy of different POCT devices varied widely, with a mean absolute relative difference (MARD) of up to 20.8 % [3]. This situation persists even though the International Consortium for Harmonization considers glucose standardization to be well-established [8], and reference systems have been described outside the United States (U.S.) [9]. A reliable glucose reference measurement procedure (RMP) could be utilized to better assess measurement accuracy and help establish and maintain metrological traceability as required by ISO 17511 [10]. Additionally, such an RMP could assist with device calibration, improvements, and monitoring of analytical performance typically conducted in standardization programs [11]. Until recently, such an RMP was not available in the U.S.

Currently, many POCT and continuous glucose monitoring devices are compared to regular clinical analyzers rather than RMPs [12]. While the importance of using RMPs has been recently highlighted [13], their use is limited. One reason for this appears to be the lack of RMPs and reference laboratory capacity [9]. To address this issue, an isotope dilution-gas chromatography-mass spectrometry (ID-GC–MS)-based candidate reference measurement procedure (cRMP) for glucose was developed for continuous operation at the Centers for Disease Control and Prevention (CDC) clinical reference laboratory.

## Materials and methods

### Chemicals and reagents

D-Glucose (Dextrose) standard reference material (SRM) 917c with a purity of 99.7 ± 0.3 % was obtained from the National Institute of Standards and Technology (NIST) (Gaithersburg, MD) for calibrator preparation. D-Glucose ^13^C_6_ with an isotopic purity of ≥ 99 atom % 13C was purchased from Sigma-Aldrich (St. Louis, MO) for use as an internal standard (IS). Hydroxylamine hydrochloride, pyridine (99.5 %, extra dry), optima grade water, ethyl acetate, ethanol, and hexanes, HPLC grade methanol and certified ACS acetic anhydride were purchased from Fisher Scientific (Waltham, MA). All additional sugars used for interference and method specificity assessment were purchased from Fisher Scientific (Waltham, MA).

### Trueness controls and serum samples

Serum-based trueness controls with certified values assigned by an established RMP were obtained from NIST [SRM 965b] (Gaithersburg, MD) and Laboratoire national de métrologie et d'essais (LNE) [CRM 101a L1, 101a L2, 301a, and 302b] (Paris, France). Pooled serum samples were obtained from Solomon Park (Burien, WA). The synthetic serum used to assess matrix effect was obtained from UTAK Laboratories (Valencia, CA). The single donor serum samples used in the comparison with point of care testing (POCT) devices were obtained from Bioreclamation IVT (Westbury, NY). These companies have institutional review board (IRB) approval to collect blood and obtained informed consent from donors. CDC’s use of the blood is consistent with the IRB approval and donor consent. No personal identifiers were provided to CDC.

### Calibration and internal standard preparation

Calibrators were prepared using D-glucose NIST SRM 917c and Optima grade water. An IS solution of ^13^C_6_ D-Glucose (Sigma-Aldrich, St. Louis, MO) was prepared in Optima grade water. For more details, refer to Table 1.

**Table 1:** Calibration and internal standard preparation for the glucose cRMP.

Stock solution concentration	Calibrator concentration, mg/dL (mmol/L)
1080 mg/dL (60 mmol/L)	378.21 (21)
	270.15 (15)
	135.08 (7.5)
	54.03 (3.0)
	27.02 (1.5)
	13.51 (0.75)

Calibrator stock and working solutions (WS) were prepared gravimetrically. An IS solution of ^13^C_6_ D-Glucose (270.15 mg/dL, [15 mmol/L]) was prepared in Optima grade water. All six calibrator working solutions and a reagent blank were processed in duplicate in the same manner as pooled serum samples in each sample batch.

### Sample preparation and gas chromatography-mass spectrometry analysis

**Fig. 1:**
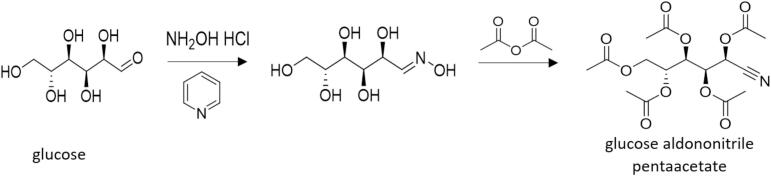
Chemical derivatization process of glucose. The CDC glucose cRMP derivatization process utilized a hydroxylamine hydrochloride solution in pyridine to convert glucose into its oxime. This oxime underwent acetylation to yield the final glucose aldononitrile pentaacetate derivative.

**Fig. 2:**
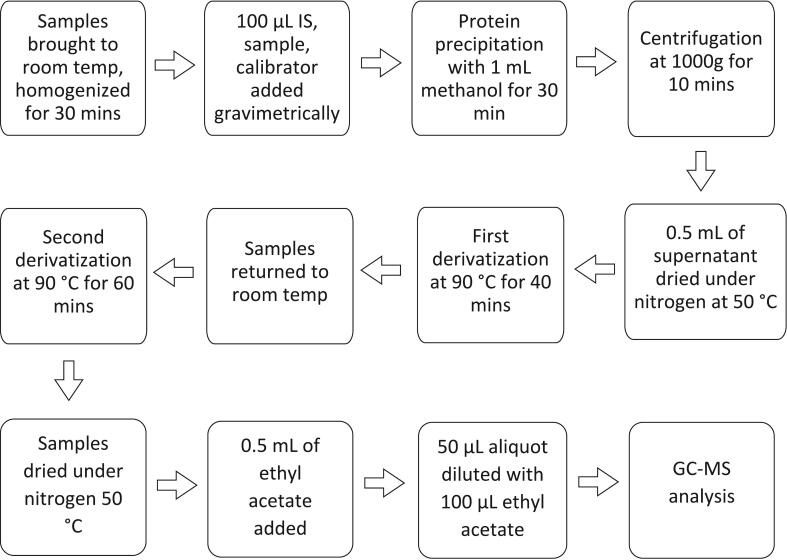
Sample preparation scheme for the glucose cRMP. All sample preparation steps that can contribute to method imprecision and bias were conducted gravimetrically. The sample preparation was adapted from previously published methods [14,15]. To release glucose from binding proteins, protein precipitation was performed, followed by centrifugation. An evaporator was used to dry the samples under nitrogen. Samples were derivatized with 150 µL of 0.2 mol/L hydroxylamine hydrochloride solution in pyridine, to convert glucose to glucose oxime. Samples were returned to room temperature, then acetylated with 200 µL of acetic anhydride to acquire the aldononitrile derivative (Fig. 1). The samples were dried under nitrogen, then brought up in 0.5 mL of ethyl acetate. A 50 µL aliquot of the glucose aldononitrile pentaacetate derivative was diluted with 100 µL of ethyl acetate for GC–MS analysis.

Sample preparation was adapted from previously published methods [14,15]. In brief, 100 µL of the trueness control, calibrator working solution (WS), and sample were gravimetrically combined with 100 µL of IS solution. To remove proteins from serum samples, protein precipitation was performed with methanol. Samples were dried under nitrogen and derivatized with 0.2 mol/L hydroxylamine hydrochloride solution in pyridine to convert the glucose into its aldononitrile derivative. Samples were returned to room temperature, and then acetylated with acetic anhydride (Fig. 1). The samples were dried under nitrogen, and then dissolved in ethyl acetate and the aliquot was further diluted with ethyl acetate prior to GC–MS analysis. More details on sample preparation and GC–MS analysis are presented in Fig. 2 and Table 2, respectively. Equipment manufacturer information and structure of the analytical series are provided in Supplemental Tables S1 and S2, respectively.

**Table 2:** GC–MS analysis parameters used for the CDC glucose cRMP.

**GC**	**System**	**Column**	**Separation method**	**Oven Temperature Gradient**	**Inlet**	**Autosampler**
	Agilent Technologies 6890 GC series	Phenomenex Zebron ZB-50 (30 m x 250 µm x 0.25 µm)	Carrier gas: Helium Flow rate: 1 mL/min Injection volume: 1 µL	Initial temp: 205 °C, for 5 min Ramp1: 15 °C/min to 235 °C Ramp2: 35 °C/min to 270 °C	Temp: 270 °C Mode: Splitless	Gerstel MPS with a cool drawer at 10 °C

**MS**	**System**	**Analysis Method**	**Ionization Mode**	**Temperatures**	**Mass transition for glucose**	**Mass transition for 13C6-glucose**
	Agilent Technologies 5975 MSD series	Selected Ion Monitoring (SIM)	Electron impact (EI) in the negative mode	Transfer line: 260 °C Source: 230 °C Quadrupole: 150 °C	*m*/*z*: 314 QI, 242 CI	*m*/*z*: 319 QI, 246 CI

Glucose analysis was carried out on an 8890/5975 GC MSD from Agilent Technologies. Sample injections were performed using a Gerstel MultiPurpose Sampler (MPS). Separation was achieved using a Phenomenex column. SIM analysis was conducted on mass to charge (*m*/*z*) 314 (quantitation ion, QI) and *m*/*z* 242 (confirmation ion, CI) for glucose and *m*/*z* 319 (QI) and *m*/*z* 246 (CI) for ^13^C_6_-glucose (Fig. 7).

Glucose was identified based on chromatographic retention time (RT) compared to a known standard (NIST 917c) and the specific *m*/*z* values of the quantitation ion (QI) and confirmation ion (CI). Peak area analysis was performed using Mass Hunter B.07.04. The ratio of the glucose QI area count to the ^13^C_6_-glucose QI area count was used to calculate concentrations. Gravimetric measurements, instead of volumetric, were used in all steps critical for method accuracy. Mass concentrations were converted to volume using density measurements of the serum. Calibrators were analyzed in duplicate, and samples were measured in four replicates for each run. Calibration curves were constructed using unweighted linear regression. Results of three independent runs (a total of twelve measurements) were used to establish reference values of serum-based materials.

### Selectivity and sensitivity

High selectivity of glucose was achieved by optimizing chromatographic separation from other saccharides. Structural analogs of galactose, mannose, xylose, arabinose, fructose, ribose, and fucose were tested to determine if they interfered with the glucose peak (Table 3). In addition, the QI/CI ratio of glucose was assessed in every sample and compared to the QI/CI ratio of matrix-free calibrators. A deviation of 20 % or more of the QI/CI ratio in the samples from the ratio observed in matrix-free calibration standards would indicate the presence of an interference in the sample [16]. The limit of detection (LOD) and the limit of quantitation (LOQ) were determined using Taylor’s method [17]. The lower limit of quantitation (LLOQ) was defined as the concentration of the lowest calibrator.

**Table 3:** List of compounds tested for interference.

Sugar	MW (g/mol)	Retention time (min)	Concentration, mg/dL (mmol/L)
D-Ribose	150.13	3.08	451.50 (30.10)
D-Arabinose	150.13	3.33	451.65 (30.11)
D-Fucose	164.16	3.37	504.46 (30.76)
D-Xylose	150.13	3.50	451.80 (30.12)
D-Mannose	180.16	6.24	542.34 (30.13)
D-glucose (NIST 917c)	180.16	6.46	542.88 (30.16)
^13^C_6_ D-glucose	186.11	6.46	279.93 (15.05)
D-Galactose	180.16	6.72	277.02 (15.39)
D-Fructose	180.16	N/A	542.16 (30.12)

Interference assessment was conducted using structural analogs of glucose. Retention times of all chromatographic peaks resolved from glucose as shown in this table. None of the tested compounds perceptibly interfered with the measurement of glucose.

### Linearity and analytical measurement range

Linearity of the measurement range was evaluated by using principles described in CLSI document, EP06-A [18]. In brief, six levels of calibrator WS were measured over the course of 20 days and the area count ratios of glucose QI to ^13^C_6_-glucose QI were plotted against the glucose concentration adjusted by specific volumes of each individual calibrator and IS. Linearity of the measurement range was assessed using residuals and linear and polynomial fitting models. The mean sum of squared residuals and the mean relative sum of squared residuals from 20 sets of calibration curves analyzed over 20 days were used to choose the best fitting regression model from among linear and polynomial models. All calculations were performed using SAS (SAS Version 9.4, SAS Institute Inc., NC, USA).

The mean slope and the mean intercept were calculated using results obtained from 15 independent calibration curves.

### Matrix effect and recovery

Matrix effect (ME) was determined in neat calibrators and serum matrices following a previously described approach [19]. Two sample matrices (synthetic serum and pooled human serum) and one set of neat samples in water were evaluated. A calibration curve ranging from 13.51−378.21 mg/dL (0.75–21 mmol/L) was prepared in each matrix in duplicate and spiked with 270.15 mg/dL (15 mmol/L) of IS solution. The calibrators in the matrix underwent the described sample preparation. The MS response (area count ratios of analyte to IS) was compared between each matrix and the neat samples prepared in water. The sample ME was calculated using the equation: ME% = B/A × 100, where “B” is the area count ratios of glucose to IS ratio obtained from samples in matrix, and “A” is the area count ratios in the matrix free samples. The slopes and R^2^ values were determined for each matrix and compared to those of a matrix-free calibration curve.

In addition, a recovery experiment was conducted by spiking 100 µL of glucose-free synthetic serum with 13.51 (0.75), 54.03 (3), 135.08 (7.5), 270.15 (15), and 378.21 (21) mg/dL (mmol/L) of glucose. Two replicates were prepared at each concentration level. Blank synthetic serum was prepared in triplicate to verify the absence of glucose. Recovery was calculated as percent difference between the expected and measured glucose concentrations.

### Accuracy, precision, and uncertainty

Accuracy was assessed by measurement of four levels of certified reference materials obtained from NIST [SRM 965b] and four levels obtained from Laboratoire national de métrologie et d'essais (LNE) [CRM 101a L1, 101a L2, 301a, and 302b]. Samples were analyzed over six independent runs with four replicates each. Significance of bias was evaluated according to NIST Special Publication 829 [20].

The within-run, between-day, and total imprecision expressed as percent coefficient of variation (%CV), were assessed in three serum pools analyzed in duplicate over 10 days. Results were analyzed according to CLSI EP10-A3-AMD [21]. In addition, results from this cRMP were compared with other laboratories through RELA, an External Quality Assessment (EQA) scheme for reference laboratories [22].

Uncertainty of the method was evaluated using the approach described in the ISO/IEC Guide to the Expression of Uncertainty in Measurement 2008 [23] (Supplemental Table S3).

### Comparison with point of care testing devices

Agreement between the glucose cRMP and two blood POCT devices (Piccolo Xpress® Analyzer [POCT A] and the Cholestech LDX® Analyzer [POCT B]) was assessed. Both POCT devices are suitable for serum glucose testing and are typically used in healthcare settings. Glucose was measured in 20 single donor serum samples covering a glucose range from 23.11 (1.28) to 376.53 (20.91) mg/dL (mmol/L). They were analyzed in two replicates over two days by the glucose cRMP and in three replicates over one day by the POCT devices. For additional information on instrument specifications, refer to Supplemental Table S4.

## Results

### Selectivity and sensitivity

**Fig. 3:**
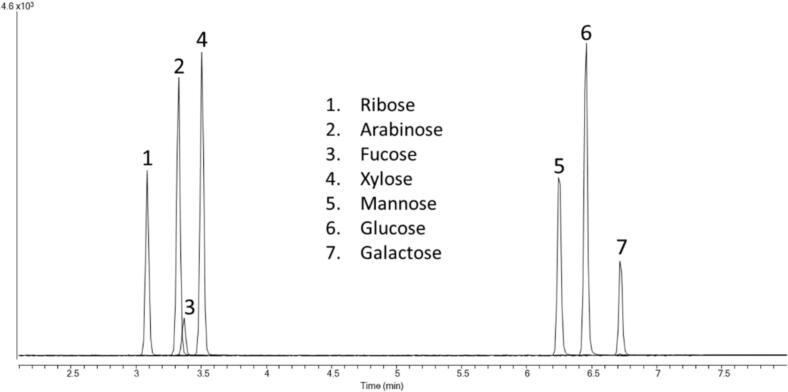
Chromatographic separation of all sugars tested in the method selectivity experiment. All aldose sugars that were examined were resolved chromatographically from glucose. This reference method is highly specific to aldose sugars by the absence of a fructose peak, which is a ketose sugar and not volatile with the derivatization approach used by this method.

Results of glucose, ^13^C_6_-glucose, galactose, mannose, xylose, arabinose, fructose, ribose and fucose mixture analysis are presented in Table 3 with their respective retention times. All aldose sugars were chromatographically resolved from glucose (Fig. 3).

**Fig. 4:**
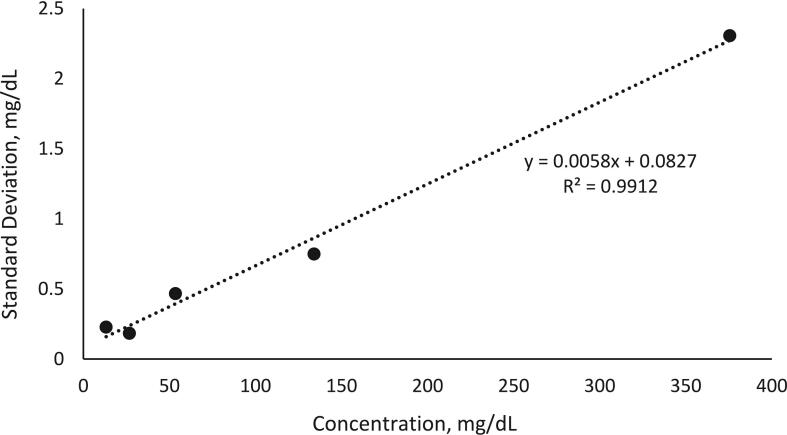
Estimation of LOD for the CDC Glucose cRMP. To determine the LOD, five levels of glucose in water (13.2 – 375.5 mg/dL) were analyzed and plotted against the standard deviation of replicate measurements of each level measured in duplicate over five days. The LOD was estimated as three times the standard deviation at the extrapolated concentration of 0.

The mean QI/CI ratio comparison in matrix and neat calibrator solutions, used to verify the absence of any potential interferences, was 1.007 (95 % CI, 0.985 to 1.028) for each calibrator. The mean QI/CI ratio of glucose for 20 serum samples was 1.011 (95 % CI, 0.994 to 1.029) (Supplemental Table S5). The LOD was 0.25 mg/dL (0.014 mmol/L) (Fig. 4) and the LOQ was 0.83 mg/dL (0.046 mmol/L). The LLOQ corresponding to the lowest calibrator concentration was 13.51 mg/dL [0.75 mmol/L].

### Linearity and analytical measurement range

The analytical measurement range (AMR) was 13.51 to 378.21 mg/dL [0.75 to 21 mmol/L], which encompasses clinically relevant concentration ranges. The calibration curve was linear throughout the AMR, and no significant polynomial relationship was observed. The mean slope of the calibration curve was 270.73 (95 % CI, 270.19 to 271.27), and the mean intercept was 0.021 (95 % CI, -0.157 to 0.199).

### Matrix effect and recovery

Matrix effect evaluation determined slopes to be 273.31, 271.96, and 271.05 in the neat, synthetic serum, and pooled serum matrices, respectively. The corresponding R^2^ values were 0.9999. The mean ME % were 99.94 (95 % CI, 99.38 to 100.50) and 98.88 (95 % CI, 97.77 to 99.99) for synthetic serum and pooled human serum, respectively.

The measurement procedure had near complete recovery with mean recoveries of 97.30 %, 100.49 %, 99.89 %, 99.99 %, and 99.96 % for the 13.51 (0.75), 54.03 (3), 135.08 (7.5), 270.15 (15), and 378.21 (21) mg/dL (mmol/L) glucose spikes, respectively.

### Accuracy, precision, and uncertainty

Accuracy of eight levels of serum reference materials was within ±0.79 % of the assigned reference value and demonstrated no significant bias (Table 4). RELA 2022 glucose challenge results for both samples A and B (as well as the entire expanded uncertainty area for reported results) reported by the CDC Laboratory were within the limit of equivalence (±3.75 %) (Supplemental Table S6) [24].

**Table 4:** Accuracy assessment of the CDC glucose cRMP.

Reference material	Reference Value, mg/dL (mmol/L)	Measured Value, mg/dL (mmol/L)	Bias, % (95 % CI)
SRM 965b, Level 1	33.08 (1.836)	33.17 (1.841)	0.28 (−0.42 to 0.97)
SRM 965b, Level 2	75.56 (4.194)	76.07 (4.222)	0.67 (0.30 to 1.05)
SRM 965b, Level 3	118.50 (6.575)	118.25 (6.563)	−0.22 (−0.47 to 0.04)
SRM 965b, Level 4	294.50 (16.35)	293.30 (16.280)	−0.41 (−0.66 to −0.15)
LNE 101a L1	74.73 (4.149)	75.06 (4.166)	0.44 (−0.32 to 1.20)
LNE 101a L2	210.12 (11.667)	209.65 (11.637)	−0.22 (−0.83 to 0.38)
LNE 301a	84.48 (4.691)	83.82 (4.65)	−0.79 (−1.48 to 0.09)
LNE 302b	128.27 (7.122)	128.02 (7.106)	−0.19 (−1.95 to 1.56)

NIST and LNE certified reference materials covering clinically relevant concentration ranges were used. The % bias ranged from −0.78% to 0.67%. Additionally, no significant bias was observed using the protocol described in NIST Special Publication 829 [20].

The inter- and intra-day imprecision were less than 0.85 % for all three levels of serum trueness controls, with a total imprecision less than 1.11 % (Table 5).

**Table 5:** Precision assessment of the CDC glucose cRMP.

	Low concentration sample	Medium concentration sample	High concentration sample
Mean concentration mg/dL (mmol/L)	33.21 (1.844)	75.84 (4.211)	292.72 (16.253)
Inter-Assay CV, % (SD, mg/dL), [mmol/L]	0.71 (0.23), [0.012]	0.68 (0.52), [0.029]	0.47 (1.36), [0.076]
Intra-Assay CV, % (SD, mg/dL), [mmol/L]	0.85 (0.28), [0.0156]	0	0.58 (1.69), [0.094]
Total CV, % (SD, mg/dL), [mmol/L]	1.11 (0.37), [0.021]	0.68 (0.52), [0.029]	0.74 (2.17), [0.120]

Inter-day, intraday, and total assay imprecision of the cRMP measurements of glucose in serum at three concentration levels. Samples were measured in duplicate over 10 days.

The relative expanded uncertainties were less than 2.24 % at each of three evaluated concentration levels and consistent throughout the entire glucose measurement range (Table 6).

**Table 6:** Estimation of expanded uncertainties of cRMP glucose measurements.

	Low sample	Medium sample	High sample
Concentration mg/dL (mmol/L)	33.17 (1.84)	76.07 (4.22)	293.30 (16.29)
Type A (%)	1.11	0.68	0.74
Type B (%)	0.18	0.18	0.18
Combined Standard Uncertainty	1.12	0.70	0.76
Coverage Factor	2	2	2
Relative Expanded Uncertainty, %	2.24	1.40	1.52

The relative expanded uncertainties were 2.24%, 1.40%, and 1.52% for low, medium, and high glucose concentration samples, respectively. Type B uncertainty made significantly smaller contribution to the uncertainty of the measurements and was constant at 0.18% for all three glucose concentration levels. The main uncertainty contribution was the Type A uncertainty obtained from repeated measurements.

### Comparison with point of care testing devices

**Fig. 5:**
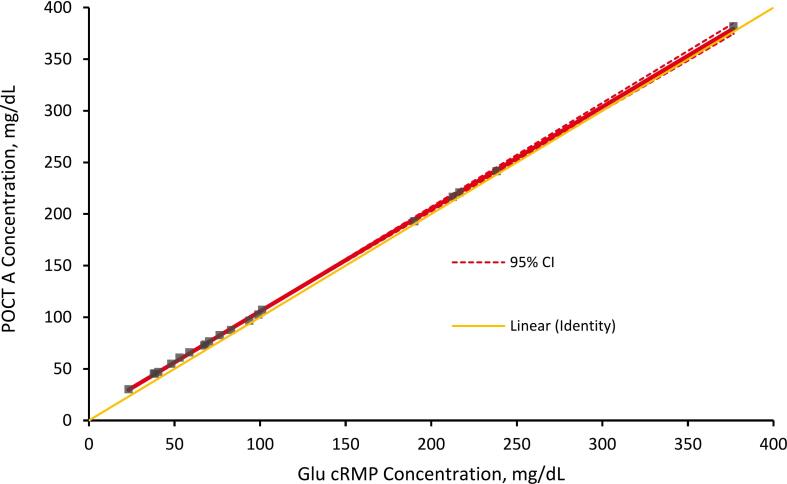
Deming regression analysis of the CDC glucose cRMP and POCT A. A set of 20 samples covering a glucose range from 23.11 (1.28) to 376.53 (20.91) mg/dL (mmol/L) was analyzed using CDC cRMP and POCT A. Concentrations determined using the cRMP were plotted against the concentrations determined by the POCT. The intercept and slope were 6.688 (95% CI 5.097 to 8.279) and 0.9904 (95% CI 0.9729 to 1.008), respectively and correlation r of 1.000. This Deming regression graph is also presented in the Supplementary Materials file with a magnified view of the hypoglycemic range (Supplemental Fig. S2).

**Fig. 6:**
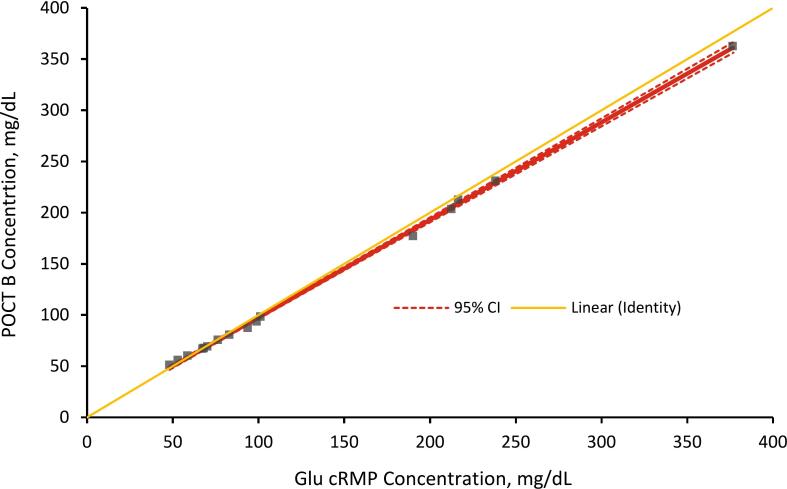
Deming regression analysis of the CDC glucose cRMP and POCT B. A set of 20 samples covering a glucose range from 23.11 (1.28) to 376.53 (20.91) mg/dL (mmol/L) was analyzed using CDC cRMP and POCT B. Concentrations determined using the cRMP were plotted against the concentrations determined by the POCT. The intercept and slope were 2.457 (95% CI 0.1285 to 4.786) and 0.9526 (95% CI 0.9348 to 0.9704), respectively and correlation r of 0.999. This Deming regression graph is also presented in the Supplementary Materials file with a magnified view of the hypoglycemic range (Supplemental Fig. S3).

Deming regression analyses comparing POCT devices to the cRMP are presented in Fig. 5, Fig. 6. For POCT A, the mean bias across all samples was 9.45 % (95 % CI, 6.00 to 12.90), and the sample-specific imprecision ranged from 0 % to 3.03 %. For POCT B, the mean bias was −1.55 % (95 % CI, −3.50 to 0.40), and the sample-specific imprecision ranged from 0 % to 4.18 %. The mean bias for samples in the hypoglycemic concentration range (glucose concentrations less than 70 mg/dL) was 16.02 % for POCT A and 2.93 % for POCT B.

**Fig. 7:**
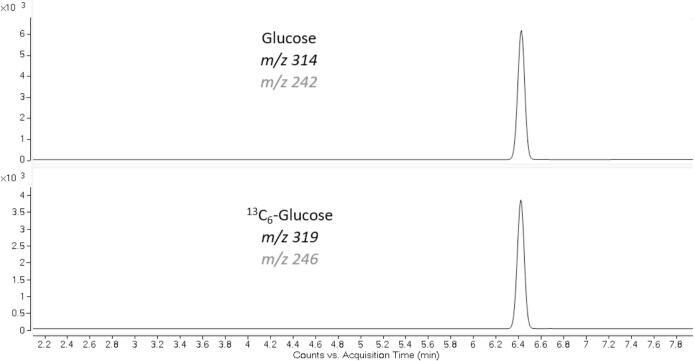
Representative selected ion monitoring traces of the quantitation and confirmation ions for glucose and ^13^C_6_-glucose. A neat sample with glucose concentration of 375.52 mg/dL (20.88 mmol/L) was used in the representative quantitation and confirmation ion chromatograms.

## Discussion

A highly accurate and precise GC–MS-based cRMP that addresses the need for an RMP for glucose in human serum was developed and validated. Traceability to SI has been established through the use of the primary reference material NIST 917c as a calibrator (Supplemental Fig. S1). The presented cRMP is intended to increase the availability of operational reference systems for glucose in the USA. The cRMP can be used as a reference point to assess routine measurements and POCT devices.

Utilizing GC–MS for the analysis of carbohydrates in blood provides strong chromatographic resolution and sensitivity [14,[25], [26], [27], [28]]. The MS in electron ionization (EI) mode produces a highly fragmented and highly reproducible spectra [29]. Commonly used derivatization approaches, such as the trimethylsilyl ether technique, can result in the formation of multiple products after derivatization [30]. To avoid this, we used the aldononitrile acetate technique to derivatize the aldose sugar, glucose, yielding only one aldononitrile acetate product [14,30].

The glucose cRMP is highly specific, as demonstrated by the interference testing experiments (Table 3, Fig. 3). Because galactose, mannose, and glucose are isomers, the MS spectra for these three monosaccharides are similar. Galactose and mannose show sufficient baseline chromatographic separation from glucose (Fig. 3). Fructose was included to demonstrate that ketose sugars do not lead to detectable derivatives. In addition, the individual QI/CI ratios of analyzed serum donor samples were within the acceptable 20 % of the mean QI/CI ratio of neat calibrators, indicating the proposed cRMP is not affected by any interferences from unknown compounds.

With the proposed cRMP, glucose concentrations can be quantitated in the clinically relevant concentration range with an accuracy and precision of ±0.79 % and less than 1.11 %, respectively. No significant ME was observed for the cRMP when slopes, R^2^, and ME% of the calibration curves in matrix were compared to those in neat solutions. The slopes of the synthetic serum and pooled serum curves were within 0.83 % of the slope of the curve prepared using neat solutions, confirming the absence of any significant ME. The extraction efficiency did not directly influence the measurement procedure’s accuracy since quantitation was based on area count ratios of glucose and IS. This is evidenced by the statistically insignificant bias of measurements for the NIST and LNE certified secondary reference materials (Table 4) and in good agreement with other mass spectrometry-based methods in the RELA survey (Supplemental Table S6). The described cRMP is a continuation of the glucose work that CDC formerly started [15] and by using gravimetric measurements, we were able to further improve the accuracy of our previous method by an average of 24 %. Applying gravimetric measurements to this cRMP also helped minimize the MU type B budget as the analytical balance contribution is negligible (Supplemental Table S3).

The developed cRMP has analytical performance that enables assay manufacturers and clinical laboratories to meet their analytical performance requirements. The criteria suggested for routine measurements include analytical imprecision of ≤2.9 %, and bias of ≤2.2 % [31]; for the criteria of POCT devices, refer to the ISO and CLSI specifications in Supplemental Table S7 [32,33]. In addition, more recently, suggestions on the measurement uncertainty (MU) performance specification for clinical laboratories and assay manufacturers, based on the clinical outcome needs, have been proposed [34,35]. The cRMP presented here has a standard MU that is in line with the recently proposed uncertainty of around 1 % for higher-order reference methods [35]. Moreover, the LLOQ of this cRMP allows measurement of samples in the hypoglycemic range.

There are three RMPs currently recognized by the Joint Committee for Traceability in Laboratory Medicine (JCTLM) for the quantitation of glucose in serum by ID-GC–MS (Supplemental Table S8) [[25], [26], [27], [28]]. The CDC cRMP demonstrates comparable accuracy, precision, and measurement uncertainty to other JCTLM-listed glucose ID-GC–MS based RMPs (Supplemental Table S8). Each evaluated individual level of reference materials covering a relevant concentration range was within 0.79 % bias, indicating that the reference method is appropriately calibrated at all concentration levels and has no sample-specific bias. The uncertainty of the cRMP is comparable to other RMPs and leaves sufficient uncertainty budget (2 % for manufacturers and clinical laboratories if 3 % allowable standard MU is used) for other components in the traceability chain related to assay manufacturers and end users to meet the maximum allowable combined standard uncertainty for a clinical sample [34]. In addition, unlike two of the JCTLM recognized RMPs for glucose, which require preparing samples for more than one day, the proposed cRMP utilizes a two-step derivatization technique and is straightforward enough to be completed in one day, thus, providing a higher throughput compared to other RMPs.

Limited data are available comparing POCT devices to RMPs. To obtain information about the comparability of POCT devices to the described cRMP, two currently available POCT devices were compared to the cRMP. The Deming regression analysis demonstrated there was no statistical difference between the POCTs and cRMP with minimal scatter (Fig. 5, Fig. 6). While both devices met the criteria for glucose POCT devices (Supplemental Table S7) [32,33], we observed a higher bias in the hypoglycemic concentration range (below 70 mg/dL). The mean bias in the hypoglycemic concentration range for POCT A was 16.02 % and for POCT B was 2.93 %. POCT A overestimated hypoglycemia for all 10 hypoglycemic samples, and while POCT B was not able to measure all 10 samples, it overestimated hypoglycemia for all samples below 60 mg/dL. One limitation of POCT B was its inability to measure glucose at acute hypoglycemic concentrations (below 50 mg/dL). The device manufacturer suggests that samples with glucose values outside the device’s measuring range of 50–500 mg/dL should be sent to a laboratory for testing. Considering that this device overestimates glucose levels below 60 mg/dL, which indicates hypoglycemia, there is a possibility that patients may not receive the immediate medical attention they need if they do have hypoglycemia. This comparative study suggests that the inaccuracy of POCT measurements in the hypoglycemic concentration range may lead to result misinterpretation and requires further improvement. Thus, this preliminary study demonstrates the need for a cRMP that can accurately and reliably assess POCT devices.

## Conclusions

We have developed an accurate and precise fit-for-purpose GC–MS cRMP for glucose in human serum. This method allows for reliable glucose measurements within the clinically relevant concentration range and meets the requirements of the CDC’s Standardization Program for glucose. The cRMP can be used to establish metrological traceability for clinical chemistry analyzer-based assays and to accurately evaluate POCT devices; it is an integral part of the glucose standardization activities at the CDC.

## Disclaimer

The findings and conclusions in this report are those of the author(s) and do not necessarily represent the official position of the Centers for Disease Control and Prevention/the Agency for Toxic Substances and Disease Registry. Use of trade names is for identification only and does not imply endorsement by the Centers for Disease Control and Prevention, the Public Health Service, and the U.S. Department of Health and Human Services.

## CRediT authorship contribution statement

**Komal Dahya:** Writing – review & editing, Writing – original draft, Validation, Investigation, Formal analysis. **Heather C. Kuiper:** Writing – review & editing, Writing – original draft, Project administration, Formal analysis. **Sarah W. Kingsley:** Writing – review & editing, Validation, Investigation. **Uliana Danilenko:** Writing – review & editing, Writing – original draft, Project administration, Formal analysis. **Hubert W. Vesper:** Writing – review & editing, Supervision.

## Declaration of competing interest

The authors declare that they have no known competing financial interests or personal relationships that could have appeared to influence the work reported in this paper.
