# Supplementary material for: Development of an isotope dilution gas chromatography − mass spectrometry candidate reference measurement procedure for glucose in human serum
Source: J Mass Spectrom Adv Clin Lab. 2025 Apr 17;36:63–72. doi: 10.1016/j.jmsacl.2025.04.005 (PMC12054013; doi:10.1016/j.jmsacl.2025.04.005)
Supplement: Supplementary Data 1 [file mmc1.docx]

Supplementary material

**Development of an isotope dilution gas chromatography - mass spectrometry candidate reference measurement procedure for glucose in human serum**

Komal Dahya^a^, Heather C. Kuiper^a^, Sarah W. Kingsley^b^, Uliana Danilenko^a^, Hubert W. Vesper^a^

^a^Division of Laboratory Sciences, National Center for Environmental Health, Centers for Disease Control and Prevention, 4770 Buford Hwy NE, Atlanta, GA, 30341

^b^Battelle Memorial Institute, 2987 Clairmont Rd, Atlanta, GA, 30329

| **Equipment/Software/Consumables** | **Manufacturer** | **Headquarters** |
| --- | --- | --- |
| 8890/5975 GC MSD | Agilent Technologies | Santa Clara, CA, USA |
| Mass Hunter B.07.04 | Agilent Technologies | Santa Clara, CA, USA |
| Density Meter | Anton Paar | Graz, Austria, USA |
| Piccolo Xpress Analyzer | Abaxis | Union City, CA, USA |
| Cholestech LDX Analyzer | Abbott | Abbott Park, IL, USA |
| MultiPurpose Sampler MPS | Gerstel | Mülheim an der Ruhr, Germany |
| GC Column | Phenomenex | Torrence, CA, USA |
| Centrifuge | Eppendorf | Hamburg, Germany |
| Evaporator | Glas-Col | Terre Haute, IN, USA |

**Supplemental Table S1** List of equipment manufactures for equipment used in the CDC Glucose cRMP.

| **Sample ID** | **Sample Type** |
| --- | --- |
| Solvent blank | Run blank |
| CC1**_**1 | Calibrator level 1 replicate 1 |
| CC1_2 | Calibrator level 1 replicate 2 |
| CC2_1 | Calibrator level 2 replicate 1 |
| CC2_2 | Calibrator level 2 replicate 2 |
| CC3_1 | Calibrator level 3 replicate 1 |
| CC3_2 | Calibrator level 3 replicate 2 |
| CC4_1 | Calibrator level 4 replicate 1 |
| CC4_2 | Calibrator level 4 replicate 2 |
| CC5_1 | Calibrator level 5 replicate 1 |
| CC5_2 | Calibrator level 5 replicate 2 |
| CC6_1 | Calibrator level 6 replicate 1 |
| CC6_2 | Calibrator level 6 replicate 2 |
| Blank**_**1 | Calibrator blank 1 |
| Blank_2 | Calibrator blank 2 |
| QC1_1 | QC sample 1 replicate 1 |
| QC1_2 | QC sample 1 replicate 2 |
| QC1_3 | QC sample 1 replicate 3 |
| QC1_4 | QC sample 1 replicate 4 |
| Solvent blank | Run blank |
| QC2_1 | QC sample 2 replicate 1 |
| QC2_2 | QC sample 2 replicate 2 |
| QC2_3 | QC sample 2 replicate 3 |
| QC2**_**4 | QC sample 2 replicate 4 |
| Solvent blank | Run blank |
| QC3_1 | QC sample 3 replicate 1 |
| QC3_2 | QC sample 3 replicate 2 |
| QC3_3 | QC sample 3 replicate 3 |
| QC3_4 | QC sample 3 replicate 4 |
| Solvent blank | Run blank |
| Sample1_1 | Sample 1 replicate 1 |
| Sample1_2 | Sample 1 replicate 2 |
| Sample1_3 | Sample 1 replicate 3 |
| Sample1_4 | Sample 1 replicate 4 |
| Solvent blank | Run blank |
| Sample2_1 | Sample 2 replicate 1 |
| Sample2_2 | Sample 2 replicate 2 |
| Sample2_3 | Sample 2 replicate 3 |
| Sample2_4 | Sample 2 replicate 4 |
| Solvent blank | Run blank |
| Sample3_1 | Sample 3 replicate 1 |
| Sample3_2 | Sample 3 replicate 2 |
| Sample3_3 | Sample 3 replicate 3 |
| Sample3_4 | Sample 3 replicate 4 |
| Solvent blank | Run blank |

**Supplemental Table S2.** Structure of the Analytical Series for the CDC Glucose cRMP.

Samples processed in one run are processed together with 6 levels of calibrator working solutions (lowest to highest concentration) in duplicate, 2 replicates of blanks, 3 levels of trueness controls in 4 replicates and 1 to 5 unknown serum samples in 4 replicates; all in the mentioned order. Depending on the number of unknown samples (which can range from 1 to 5), the total number of samples per run ranges from 30 to 46. To avoid carryover, solvent blanks are spaced between the calibrators, trueness controls and unknown samples.

| **Type B budget** | **Contribution to the standard uncertainty budget, %** |
| --- | --- |
| Purity of NIST SRM 917c | 0.15 |
| Analytical balance | negligible |
| Density meter | 0.1 |

u_typeB_ = _(_u_purity_^2^_+_u_density measurements_^2^_)_^1/2^

u_sample_= (u_typeA_^2^+u_typeB_^2^)^1/2^

**Supplemental Table S3.** Measurement uncertainty type B budget and equations for calculating type B and sample measurement uncertainties for the CDC Glucose cRMP.

The expanded uncertainty of the measurements was evaluated using the ISO Guide to the Expression of Uncertainty in Measurement 2008. The type A uncertainty was determined based on calculated imprecision of repeated measurements, and type B uncertainty budget was derived from the uncertainties in the purity of the reference material used to prepare calibrators, the inaccuracy in weighing, and in density measurements. Standard uncertainty was calculated as a square root of the sum of the squares of the type A and B uncertainties. And expanded uncertainty was calculated using a coverage factor, k = 2. Estimation of expanded uncertainties of the glucose cRMP in serum was conducted at three concentration levels (low, medium, and high), using levels 1, 2, and 4 of the NIST SRM 965b reference material. Samples were measured in four replicates over six days.

|  | **POCT A** | **POCT B** |
| --- | --- | --- |
| Sample Volume, µL | 100 | 40 |
| Testing time, min | 12 | 5 |
| Measurement range, mg/dL | 10 - 700 | 50 - 500 |

**Supplemental Table S4**. Specifications for POCT Devices used in the comparison study for the CDC Glucose cRMP.

Both POCT devices had different analytical performance specifications and operational requirements as seen above. The measurement range for POCT B presented a limitation in the study.

| A | | **Calibrator glucose QI/CI** | |  | B | **Sample glucose QI/CI** | |
| --- | --- | --- | --- | --- | --- | --- | --- |
|  |  | Replicate 1 | Replicate 2 |  |  | Day 1 | Day 2 |
| CC Level 1 | 0.941 | 0.946 |  | Sample 1 | 0.952 | 0.941 |  |
| Day 1 | CC Level 2 | 0.938 | 0.959 |  | Sample 2 | 0.957 | 0.939 |
|  | CC Level 3 | 0.949 | 0.941 |  | Sample 3 | 0.954 | 0.948 |
|  | CC Level 4 | 0.955 | 0.955 |  | Sample 4 | 0.966 | 0.963 |
|  | CC Level 5 | 0.959 | 0.957 |  | Sample 5 | 0.913 | 0.899 |
|  | CC Level 6 | 0.959 | 0.961 |  | Sample 6 | 0.988 | 0.970 |
| Day 2 | CC Level 1 | 0.972 | 0.954 |  | Sample 7 | 0.996 | 0.981 |
|  | CC Level 2 | 0.966 | 0.956 |  | Sample 8 | 0.992 | 0.983 |
|  | CC Level 3 | 0.959 | 0.964 |  | Sample 9 | 0.997 | 0.976 |
|  | CC Level 4 | 0.962 | 0.959 |  | Sample 10 | 1.000 | 0.986 |
|  | CC Level 5 | 0.964 | 0.955 |  | Sample 11 | 1.144 | 1.136 |
|  | CC Level 6 | 0.973 | 0.970 |  | Sample 12 | 1.150 | 1.134 |
| Day 3 | CC Level 1 | 0.983 | 0.958 |  | Sample 13 | 1.156 | 1.136 |
|  | CC Level 2 | 0.971 | 0.976 |  | Sample 14 | 1.160 | 1.138 |
|  | CC Level 3 | 0.979 | 0.972 |  | Sample 15 | 1.154 | 1.097 |
|  | CC Level 4 | 0.976 | 0.970 |  | Sample 16 | 0.972 | 0.978 |
|  | CC Level 5 | 0.987 | 0.987 |  | Sample 17 | 0.972 | 0.971 |
|  | CC Level 6 | 0.986 | 0.984 |  | Sample 18 | 0.975 | 0.968 |
| Day 4 | CC Level 1 | 1.128 | 1.127 |  | Sample 19 | 0.977 | 0.963 |
|  | CC Level 2 | 1.123 | 1.133 |  | Sample 20 | 0.972 | 0.973 |
|  | CC Level 3 | 1.144 | 1.138 |  |  |  |  |
|  | CC Level 4 | 1.131 | 1.142 |  |  |  |  |
|  | CC Level 5 | 1.143 | 1.136 |  |  |  |  |
|  | CC Level 6 | 1.138 | 1.145 |  |  |  |  |

**Supplemental Table S5.** Comparison of QI/CI Ratio between (A) calibrators and (B) single donor serum samples for the CDC Glucose cRMP.

Sets of 6-level calibrators were prepared as described in the ‘Calibration and internal standard preparation’ section in duplicate over 4 days, and the ratio of the glucose quantitation ion to the glucose confirmation ion were compared to those of 20 single donor serum samples prepared in duplicate over 2 analytical runs. The mean QI/CI ratio comparison in matrix and neat calibrator solutions, used to verify the absence of any potential interferences, was 1.007 (95% CI of 0.985 to 1.028) for each calibrator. The mean QI/CI ratio of glucose for 20 serum samples was 1.011 (95% CI of 0.994 to 1.029). The mean percent difference of sample to calibrator QI/CI was 0.371%.

|  | **Sample A** | **Sample B** |
| --- | --- | --- |
| Mean of 6 GC/MS labs (mmol/L) | 5.228 | 6.710 |
| % Bias | -0.35 | -0.70 |

**Supplemental Table S6**. RELA 2022 Comparison of the CDC Glucose cRMP with other Laboratories.

A total of 30 laboratories participated in the RELA 2022 glucose challenge with 6 mass spectrometry-based and 24 spectrophotometry-based assays reported. The relative bias of CDC Laboratory results compared to the mean of all six laboratories that used an ID-GC-MS method for the RELA 2022 survey are shown above.

| **Standard** | **Requirements for glucose POCT devices** | **Outcome** |
| --- | --- | --- |
| ISO^1^ | 95% of results should be within 15 mg/dL when glucose levels are less than 100 mg/dL and within 15% when levels are greater than 100 mg/dL. | Both POCT devices assessed met these requirements. |
| CLSI^2^ | 95% of results should be within 12 mg/dL when glucose levels are less than 100 mg/dL and within 12.5% when levels are greater than 100 mg/dL. |  |

**Supplemental Table S7.** ISO and CLSI requirements for POCT devices measuring glucose.

The POCT results from the preliminary comparison study between the CDC cRMP and 2 POCTs were evaluated against ISO^2^ and CLSI^3^ criteria for glucose POCT devices. While there was some bias evident in the POCT devices, both devices met the ISO 15197 and CLSI C30-A2 criteria based on the limited data acquired.

1. *ISO 15197: In vitro diagnostic test systems — Requirements for blood-glucose monitoring systems for self-testing in managing diabetes mellitus*
2. *CLSI C30-A2: Point-Of-Care Blood Glucose Testing In Acute And Chronic Care Facilities; Approved Guideline - Second Edition*

|  | **NIST** | **DGKL** | **Ghent University** | **CDC cRMP** |
| --- | --- | --- | --- | --- |
| **Method principle** | glucose is converted to glucose BBA | adapted from NIST methodology | glucose is converted to its aldononitrile acetate derivative | glucose is converted to its aldononitrile acetate derivative |
| **Measurement range** | 2 mmol/L to 20 mmol/L | 0.5 mmol/L to 20 mmol/L | 1 mmol/L to 20 mmol/L | 0.75 mmol/L to 21 mmol/L |
| **Accuracy** | N/A | N/A | bias within ±0.6% | bias within ±0.79% |
| **Precision** | CV <1.3% | N/A | CV <1.9% | CV <1.1% |
| **Expanded uncertainty (k=2)** | 0.5% to 1.5% | N/A | 1% to 2% | 1.1% to 2.24% |

**Supplemental Table S8.** Summary of analytical performance characteristics of the CDC cRMP and JCTLM listed glucose RMPs.

Current RMPs recognized by JCTLM for the quantitation of glucose in serum by ID-GC-MS include RMPs performed by NIST (Gaithersburg, MD), Deutsche Gesellschaft für Klinische Chemie und Laboratoriumsmeizin e.V. [DGKL] (Bonn, Germany), and Ghent University (Ghent, Belgium). Similar to these 3 RMPs, the CDC cRMP also utilizes the ID-GC-MS technique. The RMPs slightly differ in methodology. NIST and DGKL both perform RMPs in which glucose is converted to glucose BBA (α-D-glucofuranose cyclic 1,2:3,5-bis(butylboronate)-6-acetate) for analysis. NIST achieved high precision with the BBA method; however, the sample preparation is lengthy and requires more than 2 days to complete. NIST modified the method in 2010 to include fewer sample preparation steps, which shortened the procedure to a day and a half. The University of Ghent RMP, as well as the CDC cRMP convert glucose to its aldononitrile acetate derivative. The CDC cRMP and University of Ghent RMP are comparable.

**Procedure**

**Value assignment**

**ID-GC/MS**Glucose RMP

**Secondary Calibrator**Sera with reference values assigned by RMP

**Material**

**Calibration**

**Gravimetry**

**Primary Calibrator**NIST 917c primary material in water

**Measurand [SI units]**Serum Glucose
amount-of-substance concentration (mmol/L)

**Supplemental Figure S1**. Metrological Traceability for the CDC Glucose cRMP.

The metrological description of the measurand includes:

- the name of the quantity: *amount of substance concentration*

- the component/analyte: *Glucose*

- the biological system in which it is found: *blood serum*

- unit of measurement: *Mass fraction of glucose in serum expressed in mg/dL or mmol/L.*


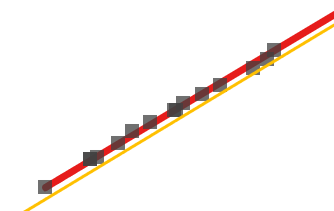


**Supplemental Figure S2**. Deming regression analysis of the CDC glucose cRMP and POCT A.

This Deming regression analysis of the CDC glucose cRMP and POCT A shows a magnified view of the hypoglycemic range to facilitate visual estimation of bias in this concentration range.


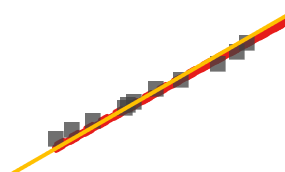


**Supplemental Figure S3**. Deming regression analysis of the CDC glucose cRMP and POCT B.

This Deming regression analysis of the CDC glucose cRMP and POCT B shows a magnified view of the hypoglycemic range to facilitate visual estimation of bias in this concentration range.
